# Characteristics and outcomes of biopsy-proven lupus nephritis in the Eastern Cape province of South Africa

**DOI:** 10.1177/09612033241281042

**Published:** 2024-09-06

**Authors:** Hanri Gerber, Robert Freercks

**Affiliations:** 1 Department of Medicine, Faculty of Health Sciences, 90475 Walter Sisulu University, Gqeberha, South Africa; 2 Division of Nephrology and Hypertension, Department of Medicine, Faculty of Health Sciences, 274490 Nelson Mandela University, Gqeberha, South Africa

**Keywords:** Systemic lupus erythematosus, Lupus, Africa, nephritis, outcomes, biopsy

## Abstract

**Objective:**

In Africa, the treatment outcomes of lupus nephritis (LN) are not well known. This is especially true in the current era where contemporary treatment options are more widely available. This retrospective study aimed to measure the outcomes of biopsy-proven LN treated at the Livingstone Tertiary Hospital (LTH) Renal Unit in Gqeberha (formerly Port Elizabeth), South Africa and to identify predictors of a poor outcome.

**Methods:**

A retrospective cohort study of 131 patients with biopsy-proven LN who had a kidney biopsy between 01 January 2012 to 31 December 2021 as identified from the biopsy register. A sub-analysis of 107 patients with proliferative and/or membranous LN was performed.

**Results:**

Mean age was 31.4 ± 12.7 years with a female predominance of 86.3%. At 6-month follow-up, 69.6% of patients had complete or partial response to treatment. This increased to 70.3% and 72.6% at 18 and 30 months, respectively. Twenty-seven patients were lost to follow-up, while 7 (5.3%) patients progressed to kidney failure (KF). There were 3 (2.3%) deaths. Predictors of poor response were an elevated baseline serum creatinine (OR = 2.53, 95% CI 0.99 – 6.52, *p* = .054), a decreased eGFR (OR = 2.92, 95% CI 0.94 – 9.09, *p* = .065) and an elevated blood pressure (OR = 6.06, 95% CI 1.11 – 33.33, *p* = .038) at the time of biopsy. Infections were the most common adverse event with 50 infections seen in 39 (29.8%) patients. Herpes viral infections were frequently noted (*n* = 12) accounting for 24.0% of all documented infections.

**Conclusion:**

Response rates were similar in this cohort when compared to other contemporary studies. Predictors of poor response were an elevated baseline serum creatinine, a decreased eGFR and an elevated blood pressure at time of the biopsy. Infections were the most common occurring adverse event, although the mortality rate remained low at 2.3%.

## Introduction

Systemic lupus erythematosus (SLE) is a complex autoimmune disease that has a heterogenous clinical presentation, ranging from mild to severe life-threatening multisystemic organ involvement, including the kidney. Up to 50% of patients with SLE experience LN, which has major implications for outcomes, since this subset of patients experience higher rates of morbidity and mortality.^1^ The most common manifestation of LN is proteinuria, but other common manifestations include reduced kidney function, haematuria and hypertension. ^1^ A kidney biopsy is recommended in all patients with suspected LN unless there is a compelling contra-indication.^2^

Previously reported predictors of a poor outcome in LN include race/ethnicity,^3,4^ hypertension,^5,6^ abnormal baseline serum creatinine,^7,8^ lack of response to induction treatment,^9,10^ proteinuria,^7,11^ LN class^12,13^ rural residence and non-adherence with medication,^14^ and non-availability of medications such as mycophenolate and chloroquine.^15^ Ethnicity has also been shown to influence the severity of SLE, the risk for the development of KF and treatment response to induction therapies. Black patients were reported to have a higher incidence of LN and a higher risk of progression to KF^16^ which may be related to the higher prevalence of APOL1 risk alleles in this population group.^17^

The outcome of patients with biopsy-proven LN in Sub-Saharan Africa (SSA) is not well known with only a few published studies to date^5,10,18–20^ and no reported studies from the Eastern Cape Region of South Africa. Furthermore, the widespread availability of mycophenolic acid derivatives and chloroquine therapy may have changed the outlook for patients with LN in the current era. This study aimed to measure the outcomes of biopsy-proven LN treated at the Renal Unit of LTH in Gqeberha, South Africa and to identify predictors of a poor outcome.

## Patients and methods

This was a retrospective cohort study of all patients with biopsy-proven LN who had a kidney biopsy between 01 January 2012 to 31 December 2021 at the Renal Unit of LTH in Gqeberha, South Africa. All patients fulfilled the modified 1997 American College of Rheumatology criteria for SLE.^21^ The patients were identified from the biopsy registry, in use since January 2012. Sociodemographic, clinical, and laboratory data at the time of the biopsy were recorded. Residential area was based on the patient’s address. Hypertension was defined as a systolic blood pressure (SBP) of >140 mmHg and/or a diastolic blood pressure (DBP) of >90 mmHg or previously diagnosed hypertension receiving treatment. Urine dipstick findings were captured and estimated glomerular filtration rate (eGFR) was calculated using the CKD-EPI formula.^22^

Histological class of LN was reported as per the pathologist, according to the Renal Pathology Society criteria.^23^ Seven patients who had been diagnosed with LN prior to 2012 were enrolled when they had a repeat kidney biopsy as part of the diagnostic work up for a flare. For patients with more than one biopsy since 2012, only the initial biopsy result was captured. Further data captured included induction regimens, maintenance treatment and adverse events including side effects and infections. Patients’ response was evaluated at 6-, 18-, and 30-months follow-up by capturing the results of repeat serum creatinine, eGFR, urine protein:creatinine ratio (uPCR) and urine dipstick findings. KDIGO guidelines were used to assess response^24^ although modified by using an early morning spot urine specimen to measure uPCR instead of a 24 h collection.^25^ Complete response was defined as a reduction in proteinuria to a uPCR <0.05 g/mmol plus stabilization or improvement in kidney function to within 10 to 15% from baseline, within 6 to 12 months of treatment initiation. Partial response was defined as a reduction in proteinuria by at least 50% and to a uPCR <0.3 g/mmol plus stabilization or improvement in kidney function to within 10 to 15% from baseline, within 6 to 12 months of treatment. No response was defined as a failure to achieve a partial or complete response within 6 to 12 months of treatment.^24^ KF was defined as an eGFR of less than 15 mL/min/1.73 m^2^ based on results traced using the National Health Laboratory System (NHLS) Labtrack system or if a patient was receiving dialysis.^26^ Patients with a complete or a partial response were grouped together as ‘response to treatment’. Patients who developed a flare of LN after a 6-month response to treatment were classified as non-response during follow-up.

Statistical analysis was conducted using SPSS version 28 (IBM, Armonk, New York). The sample was described using numerical measures of central tendency and frequency/percentage distributions. To compare continuous variables, an Independent Sample *t* test was used. If the variables did not have a normal distribution, the Mann-Whitney U Test was used. A Chi-square test or Fisher’s Exact test was used to compare categorical variables. To assess the predictors of no response in patients with biopsy-proven LN, Logistic Regression analysis was used.

Comparison of treatment response was performed in the subgroup with proliferative and/or membranous LN (Class III, IV and V) as well as for the use of CYP versus MMF as induction treatment. Patients with class III + II or class III + V were analysed together as class III. Similarly, those with class IV + V were analysed together as class IV for the outcomes analysis. Baseline characteristics identified in the literature review along with significant predictors of outcome identified on bivariate analysis (age, gender, presence of hypertension, creatinine, eGFR, uPCR, serum albumin, positive anti-dsDNA and low C3 or C4) were included in an adjusted multivariate analysis model using stepwise logistic regression. A *p*-value of less than 0.05 was considered significant.

A simplified induction regimen grouping was used for those patients who had received both MMF and CYP during the first 6-month induction phase. Thus, patients who received three or more months of intravenous or oral CYP during induction were grouped as having a CYP induction. Patients who received two or less months of CYP were grouped as having received MMF. Patients who received mycophenolic acid due to MMF intolerance were grouped with those who received MMF for the analysis. The standard of care used in treating LN in the unit can be viewed in the methods section of the supplemental material. Ethics approval was obtained from the Walter Sisulu Ethics committee prior to the initiation of the study under protocol number 091/2021.

## Results

The baseline characteristics of the overall cohort (*n* = 131) are shown in Table 1. Patient distribution in the cohort is detailed in Figure S1 of the supplemental material. The mean age was 31.4 ± 12.7 years with a female predominance of 86.3%. Only 33.0% of patients had a normal BMI, whilst 56.9% were overweight or obese. Hypertension was documented in 43.1% of patients at the time of study inclusion, with a mean systolic blood pressure of 140mmHg (range 100 to 207mmHg) whilst the mean diastolic BP was 85mmHg (range 55 to 166mmHg). At the time of biopsy, 44 patients (33.8%) had a serum creatinine of >132 µmol/L (>1.5 mg/dL). Nephrotic range proteinuria was present in 59 (46.8%) patients. Similarly, 47 patients (38.2%) had an albumin of ≤20 g/L.

**Table 1.** Baseline characteristics of overall cohort.

Baseline characteristics	All patients *n* = 131
Age ± SD (years)	31.4 ± 12.7
Gender, *n* (%)	
Male	18 (13.7)
Female	113 (86.3)
Residential area, *n* (%)	
NMBM	85 (66.4)
Country	43 (33.6)
Height ± SD, (cm)	159.9 ± 9.7
Weight ± SD, (kg)	69.8 ± 21.2
BMI ± SD, (kg/m^2^)	27.8 ± 7.3
Hypertension at study inclusion (>140/90), *n* (%)	64 (43.1)
Diabetes mellitus at study inclusion, *n* (%)	9 (6.9)
SBP ± SD, (mmHg)	140 ± 26.7
DBP ± SD, (mmHg)	85 ± 18.6
Hb ± SD, (g/dL) (range 12-15)	9.8 ± 2.4
Creatinine (IQR), (µmol/L) (range 49-90)	89 (58 – 160)
eGFR ± SD, (ml/min/1.73 m^2^)	78 ± 44
uPCR ± SD, (g/mmol creat)	0.479 ± 0.457
Low C3 (<0.90), (g/L), (*n* = 121), *n* (%)	82 (67.8)
Low C4 (<0.10), (g/L), (*n* = 103), *n* (%)	43 (41.7)
Serum albumin±SD, (g/L) (range 35-52)	23.4 ± 8.44
ANA (*n* = 124), *n* (%)	
Positive ≥1:80	120 (96.8)
Negative	1 (0.8)
Positive anti-dsDNA (*n* = 122), *n* (%)	81 (66.4)
Positive anti-Sm (*n* = 83), *n* (%)	55 (66.3)
HIV *n* = 124, *n* (%)	
Positive	8 (6.5)
Negative	116 (93.5)
Biopsy class, *n* (%)	
Class I	3 (2.3)
Class II	19 (14.5)
Class III	23 (17.6)
Class III alone	17 (13.0)
Class III + II	1 (0.8)
Class III + V	5 (3.8)
Class IV	50 (38.2)
Class IV alone	39 (29.8)
Class IV + V	11 (8.4)
Class V	34 (25.9)
Class VI	2 (1.5)

*SD* Standard deviation, *NMBM* Nelson Mandela Bay Municipality, *BMI* body mass index, *SBP* systolic blood pressure, *DBP* diastolic blood pressure, *HB* haemoglobin, *eGFR* estimated glomerular filtration rate, *uPCR* urine protein creatinine ratio, *ANA* anti-nuclear antibody, *anti-dsDNA* anti double stranded deoxyribonucleic acid, *Anti-SM* anti Smith, *HIV* human immunodeficiency virus.

Only one patient had a repetitively negative ANA. The patient had a positive ENA panel with a strongly positive anti-Sm as well as a low C3 and a C4 <0.05 g/L. The results of antibodies to other antigens were as follows: Anti-Ro (anti-SS-A) was positive in 53.5%, anti-La (anti-SS-B) in 27.1% and anti-RNP in 67.1% of patients. At the time of biopsy, one patient (0.8%) had a positive hepatitis B surface antigen and a raised hepatitis B viral load which was subsequently supressed on antiviral treatment. Of those with HIV (*n* = 8), 6 (75.0%) were virologically fully suppressed on antiretroviral therapy at the time of biopsy, with CD4 counts ranging between 88 and 644 cells/uL and a mean CD4 count of 271 ± SD 185.5 cells/uL. The HIV result was unknown for seven patients.

Proliferative (class III or IV LN) and/or membranous (class V) LN was diagnosed in 107 (81.7%) patients.

### Treatment regimens

Induction treatment consisted of various regimens which included CYP (42%), MMF (22.9%), Azathioprine (AZA) 15.2% and various other regimens/multimodal including methotrexate, dapsone, cyclosporine A, prednisone. One patient was treated with a multimodal regimen consisting of MMF, rituximab and tacrolimus.

Maintenance treatment at 18 months consisted predominantly of MMF (51.6%) or AZA (26.4%). Six patients were receiving CYP for a LN flare.

### Overall outcomes

The average length of follow-up was 34.7 ± SD 28.7 months. Follow-up data for the entire cohort is shown for each time point in Table 2. At 6-months follow-up, 69.6 % of patients had complete or partial response to treatment. This increased to 70.3% at 18-months and 72.6% at 30-months, respectively. Cumulative partial and complete response to treatment for the entire cohort over time is depicted in Figure S2 in the supplemental material. Overall, 27 patients were lost to clinical follow-up, four of whom had KF according to electronically accessible laboratory data. Follow-up data between 4 and 5 years was available for 53 patients of whom 73.6% were in complete or partial remission at the time.

**Table 2.** Outcomes of overall cohort over time.

Follow-up	At time of biopsy (*n* = 131)	6 months (*n* = 102)	18 months (*n* = 91)	30 months (*n* = 73)	
Median creatinine (IQR) (µmol/L)	89 (58 - 160)	62 (50 -83)	63 (55 – 90)	65 (53 – 98)	
eGFR±SD (ml/min/1.73 m^2^)	78.0 ± 44.0	101.1 ± 38.1	96.7 ± 39.7	95.5 ± 38.7	
uPCR±SD (g/mmol creat)	0.479 ± 0.457	0.185 ± 0.280	0.185 ± 0.302	0.203 ± 0.347	
Response, *n* (%)		71 (69.6)	64 (70.3)	53 (72.6)	
Complete		34 (33.3)	46 (50.5)	45 (61.6)	
Partial		37 (36.3)	18 (19.8)	8 (11.0)	
*Non-response, *n* (%)		32 (31.1)	27 (29.7)	20 (27.4)	
Other outcomes at time point:					Total:
Lost to follow-up		19	4	0	23
Death		3	0	0	3
Progressed to KF, no further data available		2	0	2	4
Not reached time point		1	6	10	17
Care transferred to another centre		4	3	4	11

*IQR* interquartile range*, eGFR* estimated glomerular filtration*, SD* standard deviation*, uPCR* urine protein creatinine ratio, *KF* kidney failure. *After 6 months timepoint, patients with a LN flare were also included in this category.

After achieving complete or partial response to therapy, 39 (29.8%) patients experienced one or more renal flares. Of these, 9 (6.9%) patients had two or more renal flares. KF developed in 7 (5.3%) during follow-up. At the time of biopsy, 4 (3.1%) patients were receiving dialysis for presumed acute kidney injury (AKI). Kidney replacement therapy was initiated in seven patients, two of whom remained on dialysis from the time of biopsy while no follow-up data were available for four of these patients. There was a total of 3 (2.3%) patients with confirmed deaths. The causes of death were severe community-acquired pneumonia, warfarin toxicity with rectal bleeding complicated by hospital associated pneumonia and in one case the cause of death was unknown.

On bivariate analysis of the overall cohort, the only factor associated with no response was a systolic BP >160mmHg (OR = 6.06, 95% CI 1.11 – 33.33, *p* = .038). Other factors showing a trend towards significance included: serum creatinine (OR = 2.53, 95% CI 0.99 – 6.52, *p* = .054) and an eGFR <45 mL/min/1.73 m^2^ (OR = 2.92, 95% CI 0.94 – 9.09, *p* = .065). Induction treatment with either CYP or MMF was found to be a significant predictor of achieving partial or complete response at 18-month follow-up compared to those who had an induction treatment consisting of both MMF and CYP (*n* = 9): Induction with CYP only (*n* = 40): (OR of complete or partial response = 6.89, 95% CI 1.43 – 33.18, *p* = .016). Similarly, induction with MMF only (*n* = 19): (OR for complete or partial response = 7.50, 95% CI 1.28 – 44.09, *p* = .026).

### Outcomes of proliferative (class III or IV) and/or membranous (class V) LN

Table S1 in the supplemental material shows the treatment course of 89 of the 107 patients with class III, IV or V LN for whom follow up data is available. Of these, 53 (58.9%) received induction with CYP, 26 (28.9%) with MMF and 9 (10.2%) with AZA. One patient had a multimodal induction regimen with Tacrolimus, MMF and Rituximab based on a history of recalcitrant LN. Complete/partial response rates at 6, 18 and 30 months were 70.1%, 70.9% and 75.0% respectively while 10 (12.7%) patients had experienced a flare. The majority of patients (*n* = 45, 61.6%), were maintained with MMF, whereas 21 (28.8%) patients received AZA. Of all patients in this sub-group, 9 (8.4%) patients developed KF of whom two patients had no follow-up data, and 2 (1.9%) patients died. Twenty-two patients (20.6%) were lost to follow-up.

At 6 month’s follow-up of the class III/IV/V LN subgroup (Table S2 in supplemental material), when comparing responders (*n* = 61, 70.1%) to non-responders (*n* = 26, 29.9%), the only significant predictor of non-response was the presence of hypertension at the time of biopsy (OR 2.83, *p* = .036). No significant difference in response rates was found when comparing patients who reside in remote country areas versus those who stayed nearby within Nelson Mandela Bay Metropole.

### Outcome of CYP versus MMF as induction regimen

The baseline characteristics of those patients receiving induction treatment with CYP or MMF are compared in Table 3. Although not statistically significant, those receiving CYP tended to be older and male. Significant differences between these two treatment groups included a higher uPCR, lower mean serum albumin and higher mean systolic BP in the CYP group.

**Table 3.** Baseline characteristics of those receiving MMF versus CYP.

Group divided by induction treatment	MMF (*n* = 26)	CYP (*n* = 53)	*p*-value
Age (years)±SD	27.5 ± 11	32.5 ± 13.1	0.097
Gender, *n* (%)			
Male	1 (8.3)	11 (91.7)	0.091
Female	25 (37.3)	42 (62.7)	
Hypertension at study inclusion (>140/90), *n* (%)	11 (42.3)	30 (56.6)	0.338
Diabetes mellitus at time study inclusion, *n* (%)	1 (3.8)	5 (9.4)	0.658
SBP±SD mmHg	132.1 ± 26.2	145.9 ± 27.3	0.046*
DBP±SD mmHg	82.4 ± 24.3	88.2 ± 18.7	0.270
WCC±SD (10⁹/L)	7 ± 3.4	7.8 ± 4.7	0.459
Creatinine (IQR) (µmol/L) (range 49-90)	86 (53 - 141)	101 (60 - 160)	0.491
eGFR±SD (ml/min/1.73 m^2^)	79.8 ± 43	77.3 ± 42.8	0.812
uPCR±SD (g/mmol creat)	0.413 ± 0.335	0.669 ± 0.524	0.026*
Low C3 (<0.90), (g/L), (*n* = 74), *n* (%)	17 (73.9)	34 (66.7)	0.597
Low C4 (<0.10), (g/L), (*n* = 73), *n* (%)	11 (50.0)	20 (39.2)	0.445
Serum albumin±SD (g/L) (range 35-52)	24.7 ± 7.7	20.4 ± 7.9	0.030*
Positive anti-dsDNA, (*n* = 74), *n* (%)	15 (62.5)	34 (68.0)	0.449
HIV *n* = 76, *n* (%)			
Positive		4 (7.8)	0.296
Negative	25 (100.0)	47 (92.2)	
CD4 count±SD (cells/uL)		282.8 ± 103.2	NA
HIV VL LDL or ≤20 copies/ml		4 (100.0)	NA
Biopsy class (*n* = 79), *n* (%)			
Class III	5 (19.2)	8 (15.1)	0.696
Class IV	14 (53.8)	27 (50.9)	
Class V	7 (27.0)	18 (40.0)	

*SD* standard deviation, *SBP* systolic blood pressure, *DBP* diastolic blood pressure, *WCC* white cell count, *IQR* interquartile range, *eGFR* estimated glomerular filtration rate, *uPCR* urine protein creatinine ratio, *anti-dsDNA* anti-double-stranded deoxyribonucleic acid, *HIV* human immunodeficiency virus, *VL* viral load, *LDL* lower than detectable, *MMF* mycophenolate mofetil, *CYP* cyclophosphamide. *p < .05.

At 6 months of induction therapy, there was no significant difference between the two major induction treatment modalities, with 74.0% of patients who received CYP achieving a response to treatment compared to 70.8% for those who received MMF (*p* = .459). On multivariate analysis controlling for age, gender, blood pressure, proteinuria and serum albumin, there were no significant relationships between response outcome and induction regimen. However, multivariate analysis was limited by the small cohort size and results need to be interpreted with a large degree of caution due to the grouping of patients into the predominant induction therapy used.

### Adverse events

Significant adverse events are shown in Table 4. Infections (Table 5) were the most common adverse event with 50 infections seen in 39 (29.8%) patients, none of whom were HIV positive. The most common group of infections was lower respiratory tract infection (*n* = 14) of which 8 were pneumonia, five pulmonary tuberculosis and one pneumocystis jiroveci pneumonia. Herpes viral infections were also frequently noted (*n* = 12) accounting for 24.0% of all documented infections.

**Table 4.** Significant adverse events.

Significant adverse events	Number of patients, *n* (%)
Infections	39 (29.8)
Cytopaenia’s	16 (12.2)
Gastrointestinal	13 (9.9)
Steroid induced diabetes mellitus	5 (3.8)
Avascular necrosis of the hip	2 (1.5)
Macroscopic haematuria	1 (0.8)
Premature ovarian failure	1 (0.8)

**Table 5.** Infections diagnosed in 39 patients who developed infection.

Common infections during follow-up	Number of infections, *n* = 50
Lower respiratory tract infection	
Community-acquired pneumonia	7
Hospital associated pneumonia	1
Pulmonary tuberculosis	5
Pneumocystis jiroveci	1
Herpes virus	
Herpes simplex	5
Herpes zoster	4
Varicella	3
Urinary tract infection	9
Abscesses	4
Cellulitis, furunculitis	3
Sepsis	3
Salmonella	2
*Clostridium difficile* colitis	1
Tinea	1
Candidiasis	1

## Discussion

This study examined the outcomes of biopsy-proven LN in 131 patients over a 10-year period and is the first published data on LN to emerge from the Eastern Cape Province of South Africa. The cohort was young (mean age 31.4) and predominantly female (86.3%) with 81.7% of the cohort having class III, IV or V LN. Treatment outcomes were good and comparable to other published series, with overall response rates for class III, IV and V LN of 70.1% and 70.9% at 6 and 18 months respectively. Infections were the most common adverse event, although overall mortality rates were low at 2.3%.

On average, only 13 new patients were diagnosed with LN each year while the renal unit at LTH is the only referral centre for an estimated population of ∼1.7 million people.^27^ The low incidence shown in this study’s population likely reflects poor recognition of a complex condition with a varying phenotype at a primary health care and district level; a problem which is not unique to South Africa. In a survey done in the United States of America, patients had seen a median of 3 health care providers and waited on average 3.5 years before a formal SLE diagnosis was made.^28^ The mean age at the time of kidney biopsy was 31.4 ± 12.7 years, which was similar to that reported by other studies.^18,29^

### Comorbidities

Despite the high prevalence of a BMI ≥25 (56.9% of cohort), diabetes mellitus (DM) was present in only 9 (6.9%) patients at the time of the biopsy, whilst a further 5 (3.8%) patients developed steroid induced DM during follow-up. Another South African study reported 11% of patients developing DM, whilst the Euro-Lupus trial reported 2.2%.^10,30^ In another similar SLE cohort, diabetes developed in 12.6% over a 10 year period where high dose glucocorticoids were used.^31^ The low incidence of steroid-induced DM may reflect the unit’s practice of limiting glucocorticoid use and withdrawal wherever possible (see supplementary data file for treatment protocols used). Hypertension was documented in 43.1% of patients at the time of study inclusion. Various other studies report hypertension in 31% to 55% of patients.^5,18,32^ Hypertension in the present study was shown to be a predictor of non-response to treatment, similar to a Cape Town based study.^5^ Only a small proportion of patients were HIV positive (8 patients, 6.5%). This is much lower than the national average of 13.9% and the reported rate of 29.8% in predominantly young women attending emergency units in the Eastern Cape Province where our unit is situated.^33^ Untreated HIV may be protective against the development of SLE, since CD4 T lymphocytes play an integral role in the pathophysiology of SLE development.^34^ This may explain the low prevalence of HIV in our LN cohort. Of those with HIV coinfection, four received induction therapy with CYP; all were virologically suppressed, and none had any documented infections during follow-up. At 30 months follow-up, three patients (75%) had complete/partial response to treatment, whilst one patient was lost to follow-up. There is limited data guiding immunosuppressive treatment strategies in people living with HIV. In a study by Hax et al., two people living with HIV in their cohort received CYP for LN with good response and no major infections were reported.^35^

### Biopsy class and response outcome

In this study, 107 patients (81.7%) had class III, IV or V LN with a complete or partial remission rate of 70.1% after induction therapy, which increased to 70.9% at 18 months. The treatment response rate was comparable to other studies and is shown in Table 6 below.^9,18,36^

**Table 6.** Comparison of outcomes of lupus nephritis in published studies.

Study and year	Country	*N*	Class of LN	Complete remission	ESKD	Deaths
Almalki et al., 2019^43^	Saudi Arabia	96	III,IV,Mixed V+III/IV	39%	10 (11.5%)	5 (5.2%)
Arends et al., 2014^44^	Netherlands	71	III,IV, mixed V+III/IV	64.6%	1 (1.4%)	2 (2.8%)
Ayodele et al., 2012^10^	South Africa	66	III,IV	26 (39%)	20 (30%)	26 (39%)
Ayodele et al., 2010^5^	South Africa	105	II-VI		7 (6.7%)	32 (30%)
Fernandes das Neves et al., 2015^45^	United Kingdom	105	III-V, mixed V+III/IV			14 (13.3%)
Hui et al., 2012^36^	United Kingdom	61	III,IV, mixed V+III/IV	35.20%	5 (8.2%)	3 (4.9%)
Mahmoud et al., 2015^9^	Egypt	135	II-VI	Not stated	3 (2.2%)	17 (12.6%)
Mody et al., 2018^18^	South Africa	87	III,IV, mixed V+III/IV	40%	6 (6.8%)	4 (4.5%)
Norby et al., 2017^13^	Norway	178	I-VI	Not stated	36 (20.2%)	36 (20.2%)
Okpechi et al., 2012^19^	South Africa	42	V,V+III,V+VI	Not stated		Not stated
Pakfetrat et al., 2017^11^	Iran	80	II,III,IV,V	52 (67.5%)	6 (7.8%)	3 (3.7%)
Pinto-Penaranda et al., 2015^46^	Colombia	149	III,IV,Mixed V+III/IV	39%		
Siso et al., 2010^47^	Spain	190	I,II,III,IV,V,VI,II+III,II+VI,III+V	74%	18 (9%)	19 (10%)
Yang et al., 2015^7^	China	1814	II,III,III+V,IV,IV+V,V,VI	Not stated	201 (11.1%)	Not stated

### Predictors of response

Induction treatment with either CYP or MMF alone was found to be a predictor of complete or partial remission at 18 months when compared to the group who had altered induction treatment with a combination of CYP and MMF of varying duration. This most likely reflects a more complicated or resistant course necessitating treatment change either due to side effects or non-response. Other factors associated with non-response included lower kidney function and higher blood pressure, likely reflecting more severe disease. We compared the two main induction regimens of CYP and MMF. On bivariate analysis, those in the CYP group appeared to have more severe disease with higher systolic blood pressure (145.9 vs 132.1 mmHg), higher uPCR (0.669 vs 0.413 g/mmol) and lower serum albumin (20.4 vs 24.7 g/L) reflecting the unit’s preference for CYP in those with severe disease. On multivariate analysis controlling for disease severity, there was no statistically significant difference in response rates between CYP and MMF when used for induction, with a similar outcome response at 6 months (74.0% vs 70.8%, *p* = .459). Although this is similar to the seminal ALMS trial,^29^ our results need to be interpreted with a large degree of caution due to the small sample size, the manual assignment of treatment groups, periods of treatment crossover and the retrospective nature of the study. The relatively frequent use of CYP in our unit also reflects its low cost, ease of administration and guaranteed patient adherence during the induction phase. Adherence to therapy is a well-known treatment challenge in lupus cohorts.^37^ This is especially so with MMF which has frequent gastrointestinal side effects and requires twice per day dosing.^38^

### Kidney failure and mortality

Overall, 11 patients (8.4%) had or developed KF, which is comparable to two other similar studies from China and South Africa reporting rates of 6.9% and 11.1% respectively.^7,18^ Another South African study reported a KF rate of 30%.^10^ A total of 3 (2.3%) patients were confirmed to have died during the follow-up period, although the true figure may be higher due to those lost to follow-up. Of the 20 patients that were lost to follow-up, 4 (3.1%) were noted to have kidney failure according to the NHLS Labtrack system after which no further data was available to establish their outcome. Since the Renal Unit at LTH is the only public sector facility in the region to offer dialysis, it may be reasonably assumed that these patients have since died. That being the case, our reported mortality rate would increase to 5.3%, which remains in line with other reported contemporary cohorts and well below an earlier South African study where a mortality rate of 39% (the majority due to sepsis) was reported. In that study oral CYP was predominantly used with much higher cumulative doses.^10^ Improved outcomes in LN have been noted worldwide and may reflect improved understanding of the disease as well as expanded and optimised treatment regimens.^39^ In the seminal multicentre ALMS study comparing CYP and MMF, 4.9% of the MMF group and 2.8% of the CYP group died during the study.^29^

### Other adverse events

Infections were the most common adverse event noted in this cohort, although the mortality rate remained low. Tuberculosis was diagnosed in 5 (3.8%) patients which is significantly lower than the 14.1% development of tuberculosis reported in a large South African cohort of patients with SLE and long term follow-up.^40^ This supports the use of universal isoniazid prophylaxis as used in our cohort, although a longer duration of prophylaxis may need to be considered. It was noted that a large number of patients developed herpes viral infections (9.2%). Multiple other studies have similarly shown a high incidence of herpes viral infections.^41^ There are no clear guidelines or data available to support the use of antiviral prophylaxis and further research is needed to evaluate the benefit of this.^42^ Despite the relatively frequent use of CYP, premature ovarian failure was an uncommon occurrence, being seen in only one patient.

### Strengths and limitations

A strength of this study is that it spans an era where internationally accepted treatment modalities have become universally available. MMF was previously not a widely used drug, due to limited access especially in the public health sector. In addition, the Renal Unit at LTH uses chloroquine in all patients with LN, unless there is a compelling contra-indication.

One of the limitations was the retrospective nature of the study. Some baseline parameters were not available for every patient and not all folders were available. These patients formed part of the group that were noted as lost to follow-up/unknown and only their baseline findings that were available at the time of biopsy were recorded. Twenty-seven (20.6%) patients were lost to follow-up. Interestingly, 9 (33.3%) had class II LN on biopsy. It is possible that patients have sought medical care in the private sector, moved to another district/province or may have died. Alternatively, they may have had mild disease not requiring intense immunosuppressive therapy. While race/ethnicity are associated with outcomes in LN, this parameter was not self-reported and hence could not be captured. However, in an unpublished biopsy audit from the same unit and cohort, the ethnic representation was 57.7% Black African, 34.6% mixed ancestry and 7.7% White and it is likely that our cohort will be similarly represented. Because this was not a clinical trial, decisions regarding induction and maintenance therapy were made by the treating clinician and were not randomly assigned. This led to frequent dosage adjustments and modality switches as side effects were experienced or if there was a lack of response. Therefore, no firm conclusions can be drawn regarding the relative therapies used. Finally, the relatively small cohort size limited the use of multivariate analysis.

## Conclusion

In conclusion, this study included 131 patients with biopsy-proven LN and demonstrated response rates similar to other contemporary studies. The only predictor of no response to treatment was an elevated blood pressure at time of the biopsy. Infections were the most common occurring adverse event, although the mortality rate remained low at 2.3%. Further research is required to ascertain the role for antiviral chemoprophylaxis in current treatment protocol.

## Supplemental Material

Supplemental Material - Characteristics and outcomes of biopsy-proven lupus nephritis in the Eastern Cape province of South Africa Supplemental Material for CCharacteristics and outcomes of biopsy-proven lupus nephritis in the Eastern Cape province of South Africa by Hanri Gerber and Robert Freercks in Lupus
