# Supplementary material for: Characteristics and outcomes of biopsy-proven lupus nephritis in the Eastern Cape province of South Africa
Source: Lupus. 2024 Sep 6;33(12):1289–98. doi: 10.1177/09612033241281042 (PMC11437693; doi:10.1177/09612033241281042)
Supplement: Supplemental Material - Characteristics and outcomes of biopsy-proven lupus nephritis in the Eastern Cape province of South Africa [file sj-pdf-1-lup-10.1177_09612033241281042.pdf]

Supplemental material

Methods: Standard of care used for treatment of LN in LTH Renal Unit

The Renal Unit at Livingstone Tertiary Hospital uses chemoprophylaxis for Mycobacterium Tuberculosis (isoniazid and pyridoxine) and Pneumocystis Jiroveci (cotrimoxazole) in all patients during induction therapy, as well as universal long-term chloroquine use unless contraindicated. Prophylaxis against gastrointestinal ulceration is used (proton pump inhibitors) while corticosteroid (prednisone) doses exceed 7.5mg per day. Calcium and vitamin D supplementation are routinely given during treatment with corticosteroids. Vaccination against pneumococcus, influenza and COVID is encouraged, although is not always available.

In those with more severe class III/V LN, or with class IV LN, cyclophosphamide (CYP) or mycophenolate mofetil (MMF) are used for a 6-month induction treatment period. The treating nephrologist determines which agent to use based on patient preference, age and severity of disease with CYP being preferred for crescentic nephritis and where fertility concerns are less relevant. When used, CYP is given as a monthly intravenous infusion of 750mg/m^2 but reduced to 500mg/m^2 for those with an eGFR <30ml/min or age >65 years. CYP or MMF is discontinued after 6 months' induction or on achievement of complete remission. A record is kept of the total cyclophosphamide dose infused over patients' lifetime and this is generally limited to a maximum of 15g. During induction, corticosteroids are also administered. In class III or IV LN or those with class V and nephrotic range proteinuria, corticosteroids are initially given as an intravenous infusion of methylprednisolone of 500mg daily for 3 days, which is then followed by a tapering dose of oral prednisone beginning with 0.5mg/kg/day and aiming to reduce to ≤0.15mg/kg/day by 3 months. For mild class III or class V LN, azathioprine is frequently used as induction and maintenance therapy. Corticosteroids are routinely discontinued by 6 months unless lupus activity requires continuation.

After 6 months of induction therapy is completed or on attainment of complete remission, MMF is routinely used for maintenance therapy unless not tolerated in which case azathioprine will usually be used

Supplemental tables and figures

Table S1: Induction regimens used and outcomes of proliferative and membranous lupus nephritis.

Proliferative and Membranous lupus nephritis, n (%)		
		N=107
Class	III	23 (21.5)
	IV	50 (46.7)
	V	34 (31.8)
Induction	Cyclophosphamide	53 (58.9)
	MMF/Mycophenolic acid	26 (28.9)
	Azathioprine	9 (10.2)
	Other	2 (2.2)
Response 6 months	Complete/ Partial	61 (70.1)
	Non-response	26 (29.9)
Maintenance 18 months	MMF/Mycophenolic acid	45 (61.6)
	Azathioprine	21 (28.8)
	Other	7 (9.6)
Response 18 months	Complete/ Partial	56 (70.9)
	Flare	10 (12.7)
	Non-response	13 (16.4)
Maintenance 30 months	MMF/Mycophenolic acid	31 (56.4)
	Azathioprine	17 (30.9)
	Other	7 (12.7)
Response 30 months	Complete/ Partial	48 (75.0)
	Flare	6 (9.4)
	Non-response	10 (15.6)
KF		9 (8.4)
Lost to follow-up		22 (20.6)
Death		2 (1.9)

KF kidney failure, *MMF* mycophenolate mofetil.

Table S2: Overall predictors of outcome in proliferative and membranous lupus nephritis subgroup, N = 87.

Variable	Responders (n= 61)	Non-responders (n=26)	p-value	OR
Age (years) ± SD	31.3 ± 12.8	30.7 ± 12.2	0.283	1.03
Gender, n (%)			0.551	0.62
Male	7 (11.5)	6 (23.1)		
Female	54 (88.5)	20 (76.9)		
Hypertension, n (%)			0.036	2.83
Yes	27 (44.3)	18 (69.2)		
No	34 (55.7)	8 (30.8)		
Median baseline creatinine (IQR) (umol/L) (range 49-90)	86 (53 - 120)	103 (64 - 159)	0.317	1.00
eGFR (ml/min/1.73m²)	84.8	76.2	0.254	1.01
uPCR (g/mmol creat)	0.517	0.543	0.450	1.81
Serum albumin (g/L) (range 35-52)	23.1	22.6	0.464	0.97
Positive anti-dsDNA antibody, n (%)	40 (69.0)	14 (63.6)	0.615	
Low C3 (<0.9), n (%)	39 (70.9)	16 (64.0)	0.581	0.66
Low C4 (<0.1), n (%))	20 (37.7)	8 (32.0)	0.400	0.54

IQR interquartile range, *eGFR* estimated glomerular filtration rate, *uPCR* urine protein creatinine ratio, *anti-dsDNA* anti-double stranded deoxyribonucleic acid.

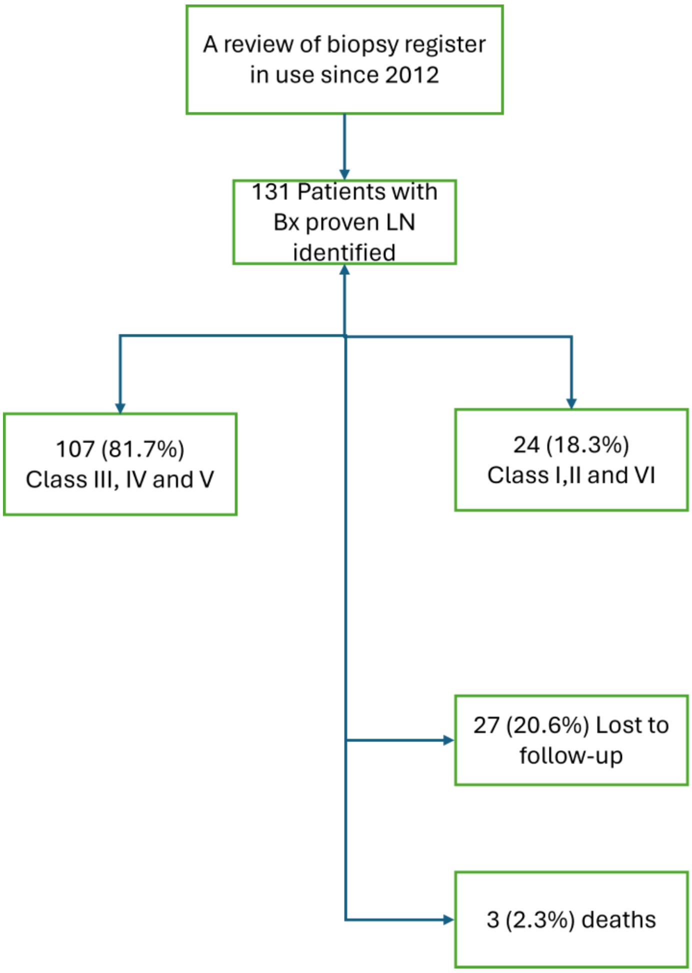

Figure S1: Flow chart of patient distribution

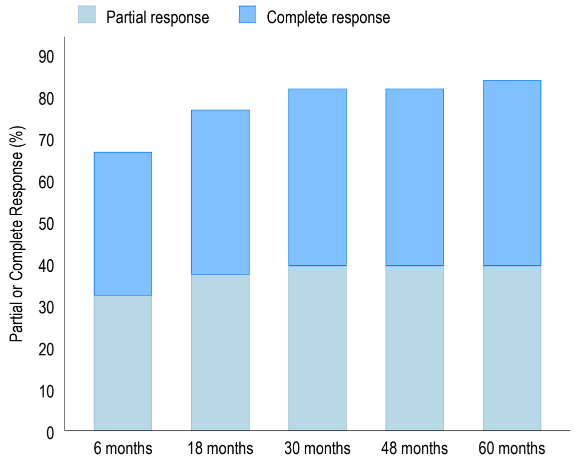

Figure S2: Graph depicting cumulative partial and complete response to treatment for the entire cohort over time
